# Supplementary material for: Proteomic analysis defines kinase taxonomies specific for subtypes of breast cancer
Source: Oncotarget. 2018 Jan 29;9(21):15480–97. doi: 10.18632/oncotarget.24337 (PMC5884642; doi:10.18632/oncotarget.24337)
Supplement: Supplementary file 1 [file oncotarget-09-15480-s001.pdf]

Proteomic analysis defines kinase taxonomies specific for subtypes of breast cancer

SUPPLEMENTARY MATERIALS

Supplementary Table 1: Kinases bound to individual beads. See Supplementary_Table_1

Supplementary Table 2: Understudied kinases list. See Supplementary_Table_2

Supplementary Table 3: Kinases bound uniquely within a subtype

Basal-like	Claudin-low	HER2-enriched	Luminal
DAPK2	ADCK4	CDKL3	BLK
DUSTY	BRD2	CSKP	ERBB4
MAST1	CD11A	DDR2	INSRR
MAST2	CTRO	ERBB2	KAPCG
NEK11	HIPK3	HXK1	LRRK2
NRK	KPCG	JAK3	M3K10
	M3K7	K6PF	MK15
	MK07	KPCB	VGFR3
	MYLK	M3K13	
	NEK4	MAK	
	NEK6	PCKGM	
	NEK7	RIPK3	
	PAK7	TESK2	
	PASK	TRIO	
	PGFRA		
	SGK1		
	STK33		
	STK39		
	UHMK1		

Supplementary Table 4: Kinases within subnetworks. See Supplementary_Table_4

Supplementary Data File 1: Baseline MIB-binding data matrix. See Supplementary_Data_File_1

Supplementary Data File 2: Matrix of RSEM values across cell lines. See Supplementary_Data_File_2

Supplementary Data File 3: MIB-binding response data matrix. See Supplementary_Data_File_3

Supplementary Data File 4: Unique kinases by subtype. See Supplementary_Data_File_4

Supplementary Data File 5: Kinase subnetworks. See Supplementary_Data_File_5
